# Pannexin 1 regulates postnatal neural stem and progenitor cell proliferation

**DOI:** 10.1186/1749-8104-7-11

**Published:** 2012-07-04

**Authors:** Leigh E Wicki-Stordeur, Adrian D Dzugalo, Rose M Swansburg, Jocelyne M Suits, Leigh Anne Swayne

**Affiliations:** 1 Division of Medical Sciences, Island Medical Program, University of Victoria, Victoria, British Columbia, Canada; 2 Department of Biology, University of Victoria, Victoria, British Columbia, Canada; 3 Department of Biochemistry and Microbiology, University of Victoria, Victoria, British Columbia, Canada; 4 Department of Cellular and Physiological Sciences, University of British Columbia, Vancouver, British Columbia, Canada

**Keywords:** Postnatal neurogenesis, Pannexin 1, Neural stem and progenitor cells, Cell proliferation, ATP release

## Abstract

**Background:**

Pannexin 1 forms ion and metabolite permeable hexameric channels and is abundantly expressed in the brain. After discovering pannexin 1 expression in postnatal neural stem and progenitor cells we sought to elucidate its functional role in neuronal development.

**Results:**

We detected pannexin 1 in neural stem and progenitor cells *in vitro* and *in vivo*. We manipulated pannexin 1 expression and activity in Neuro2a neuroblastoma cells and primary postnatal neurosphere cultures to demonstrate that pannexin 1 regulates neural stem and progenitor cell proliferation likely through the release of adenosine triphosphate (ATP).

**Conclusions:**

Permeable to ATP, a potent autocrine/paracine signaling metabolite, pannexin 1 channels are ideally suited to influence the behavior of neural stem and progenitor cells. Here we demonstrate they play a robust role in the regulation of neural stem and progenitor cell proliferation. Endogenous postnatal neural stem and progenitor cells are crucial for normal brain health, and their numbers decline with age. Furthermore, these special cells are highly responsive to neurological injury and disease, and are gaining attention as putative targets for brain repair. Therefore, understanding the fundamental role of pannexin 1 channels in neural stem and progenitor cells is of critical importance for brain health and disease.

## Background

**Figure 1:**
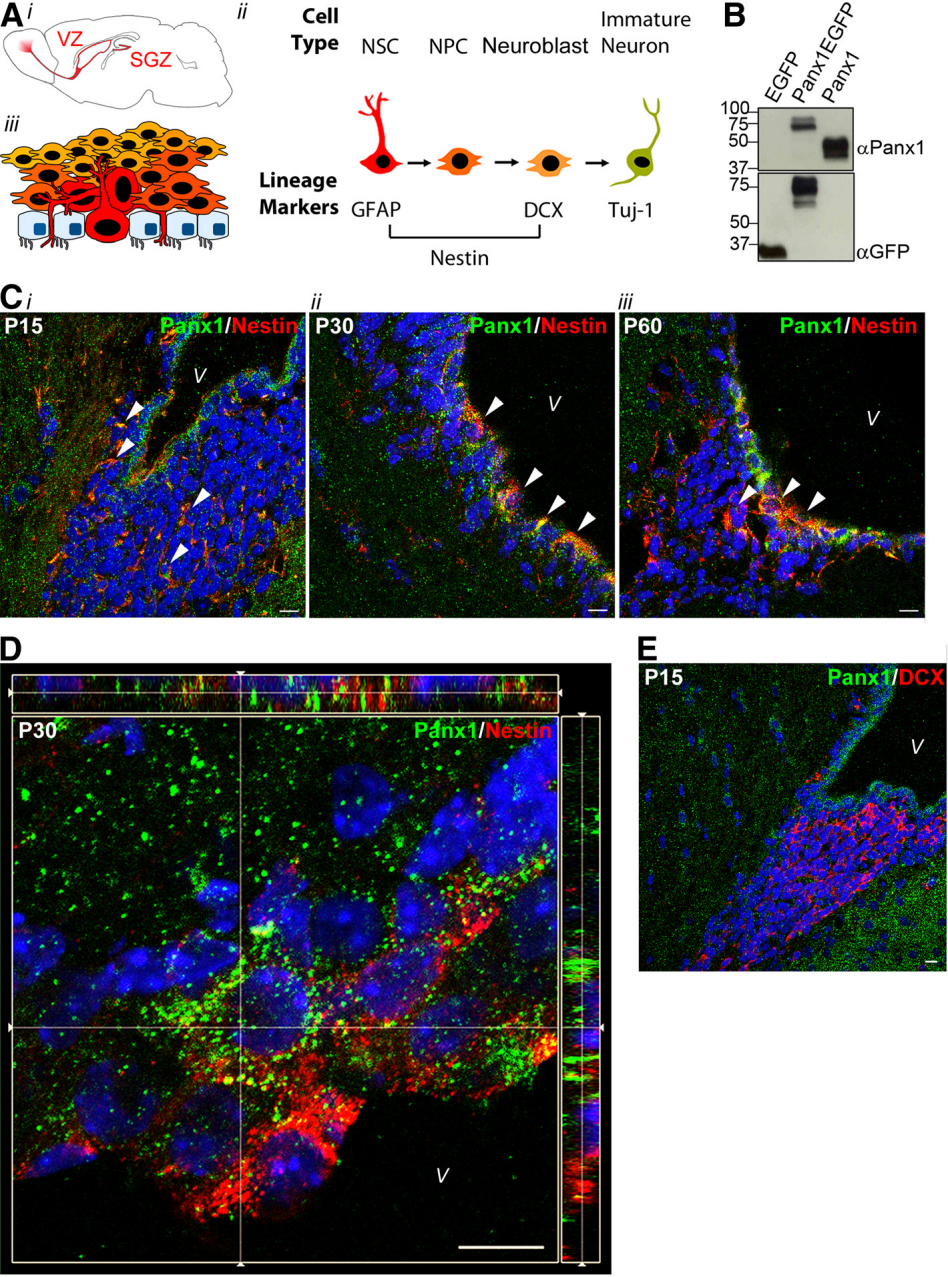
**Panx1 is expressed in nestin-positive periventricular NSC/NPCs throughout postnatal development* in vivo.*** (**A**) Mouse brain schematic with the neurogenic ventricular zone (VZ) and subgranular zone (SGZ) regions highlighted (*i*). Cell types throughout neurogenesis and the lineage markers expressed at each stage. Relatively quiescent NSCs become highly proliferative NPCs, and neuronally committed migratory neuroblasts before exiting the cell cycle as immature neurons (*ii*). Schematic of cell types in the periventricular region. The ependymal cell layer is interdigitated by thin processes or whole luminal surfaces of the radial glia-like NSCs (*iii*). This figure is a partial reproduction of another figure created by the same authors that appears in [2] and is reproduced with permission. (**B**) Western blot of lysates from HEK293T cells overexpressing EGFP, Panx1EGFP, or Panx1, showing specificity of the Panx1 Cterm antibody. (**C**) Confocal images of murine brain slices at the periventricular region. Nestin-positive NSCs exhibit high levels of Panx1 co-expression in postnatal day 15 (P15) (*i*), P30 (*ii*) and P60 (*iii*) brains. Arrows indicate areas of co-expression. (**D**) Image from a confocal z-stack with orthogonal side-views of a P30 periventricular region. Panx1 expression is noted in ependymal/sub-ependymal layers. (**E**) Confocal image from a P15 periventricular region. Panx1 is absent from DCX-positive neuroblasts. Hoechst 33342 was used as a nuclear counterstain in C-E. All scalebars 10 μm. DCX, doublecortin; NPC, neural progenitor cells; NSC, neural stem cells; Panx1, pannexin 1; V, ventricle.

The majority of neurons are born embryonically, however neurogenesis continues in the postnatal brain throughout life (reviewed in [1]). Postnatal neurogenesis is a complex, multi-step developmental process that includes cell behaviors such as proliferation, differentiation and migration, and involves several distinct cell types. These stem cell behaviors are guided, in part, by extracellular stimuli unique to their specialized microenvironments or ‘niches’: the subgranular zone (SGZ) of the dentate gyrus and the ventricular zone (VZ) of the lateral ventricles (Figure 1A).

Postnatal NSCs in the VZ generate large numbers of olfactory bulb interneurons. The distinct neural stem and progenitor cell (NSC/NPC) types implicated in this neurogenic process are characterized by their morphology and differential expression of lineage markers (Figure 1A). Radial glia-like cells (known as type B cells in VZ) express the filament markers nestin and glial fibrillary acidic protein (GFAP) (as well as several other lineage markers). Electron microscopy has shown that the ependymal layer lining the ventricles is not entirely contiguous and that type B cells make direct contact with the ventricle either via a large luminal surface or a thin cellular process (Figure 1A) [3-8]. These cells subsequently generate highly proliferative intermediate, transit-amplifying NPCs (C cells) that lose GFAP expression. Finally, further differentiation gives rise to migratory neuroblasts (A cells), which are committed to a neuronal lineage and express doublecortin (DCX). Following exit of the cell cycle, the immature neurons express class III beta tubulin, also known as Tuj-1.

Pannexins (Panx) are four-pass transmembrane proteins that oligomerize to form large pore ion and metabolite-permeable channels. Building on accumulating evidence that the coordinated activity of ion channels plays a major role in all aspects of NSC/NPC biology [9], we recently found that intracellular Panx2 modulates neuronal differentiation by impacting on neurite outgrowth and the timing of NSC/NPC commitment to a neuronal lineage [10].

Another Panx family member, Panx1, is also widely expressed in the brain as well as being found in the periphery [11-15]. Panx1 forms large hexameric pores at the plasma membrane and/or at the endoplasmic reticulum membrane, depending on the cell type, and is permeable to ions and ATP in neurons, astrocytes and microglia [16-21]. Purinergic nucleotides act as proliferation signals for NSC/NPCs, serving as negative regulators of terminal neuronal differentiation [22-25]. NSC/NPCs express P2Y and P2X7 receptors and release ATP in episodic burst events, which act in an autocrine and paracrine manner to induce proliferation and negatively regulate differentiation. We have discovered Panx1 expression in postnatal NSC/NPCs. As large pore channels that mediate ATP release, we hypothesized that Panx1 channels might be positively associated with NSC/NPC proliferation.

Here we combine studies in the Neuro-2a (N2a) murine neuroblastoma-derived cell line as model NSC/NPCs, and VZ NSC/NPCs *in vitro* and *in vivo*, to elucidate the role of Panx1 in postnatal neuronal development. We detected Panx1 in nestin-positive and GFAP-positive NSC/NPCs and Tuj1-positive immature neurons, but not in intermediate DCX-positive neuronally committed neuroblasts *in vitro* and *in vivo*. Panx1 mediated ATP release, and blocking P2 purinergic receptors reduced cell proliferation, suggesting a possible role for Panx1 in regulating cell proliferation. Finally, by modulating Panx1 expression and activity in N2a cells and VZ neurosphere cultures, we demonstrated that Panx1 regulates NSC/NPC proliferation.

## Results

### Panx1 is expressed in periventricular NSC/NPCs

Using confocal immunofluorescence microscopy, we examined coronal cryosections of the postnatal periventricular area ranging from postnatal day 15 (P15) to P60 with lineage-marker antibodies (Figure 1C) and for Panx1 expression with an antibody (anti-Panx1 Cterm rabbit polyclonal, Invitrogen/Life Technologies, Burlington, Ontario, Canada) that we had previously characterized by immunoblotting of lysates from human-embyonic kidney (HEK) 293T cells transfected with Panx1, Panx1EGFP and EGFP (Figure 1B). Panx1 was detected in a sub-population of nestin-positive/GFAP-positive type B cells and nestin-positive type C cells in the ependymal/sub-ependymal layer of both the dorsal (Figure 1C, D) and ventral (not shown) aspects of the lateral ventricles. Panx1 was absent from DCX-positive committed neuroblasts (Figure 1E). These results suggest that Panx1 is expressed in periventricular NSC/NPCs and is lost upon neuronal commitment.

### Panx1 mediates ATP release from NSC/NPCs

**Figure 2:**
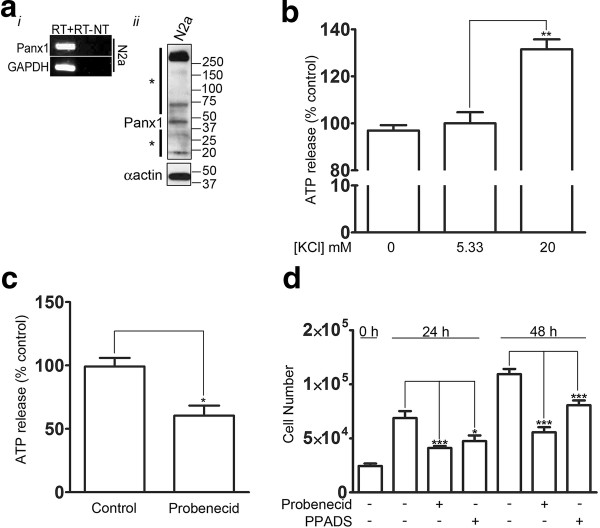
**Panx1 blockage decreases ATP release from N2a cells and blocking P2 receptors reduces cell proliferation.** (**A**) Panx1 is endogenously expressed in N2a cells, as demonstrated by RT-PCR (*i*) and western blot analysis (*ii*). To the left of the western blot, the asterisk (*) above the arrow denotes high-molecular weight species likely representing various Panx1 oligomeric forms, while the asterisk below the arrow denotes low molecular weight Panx1 cleavage products consistent in size with those reported in the literature [16]. (**B**) ATP release from N2a cells was stimulated upon treatment with 20 mM KCl buffer compared to control (5.33 mM) and 0 mM KCl conditions ((**) *P* < 0.01 for one-way ANOVA with *post-hoc* Tukey’s multiple comparison test, N = 3). (**C**) Blockage of Panx1 channels with 0.5 mM probenecid significantly decreases stimulated ATP release from N2a cells compared to controls (60.50 ± 7.913% and 99.17 ± 6.831%, respectively, N = 5, *P* = 0.0159 for non-parametric *t* test). (**D**) N2a cell numbers were quantified at 0, 24 and 48 hours for cells treated with 30 μM PPADS, 1 mM probenecid, or vehicle control. PPADS and probenecid treatments significantly reduced N2a numbers at 24 and 48 hours ((*) *P* < 0.05 and (***) *P* <0.001 for one-way ANOVA with *post-hoc* Tukey’s multiple comparison test, N = 3). ANOVA, analysis of variance; Panx, pannexin1, PPADS, pyridoxalphosphate-6-azophenyl-2′,4′-disulfonic acid.

To further study the properties and cell biology of Panx1 in neuronal development we moved to *in vitro* culture models. We first employed the N2a cell line to examine the ability of Panx1 expression to influence ATP release from NPCs and to confirm the effects of blocking P2 receptors on NPC proliferation. The N2a cell line, derived from a murine neuroblastoma, is a widely used NPC model as these cells can be differentiated in the presence of retinoic acid and low serum [10,26,27]. N2a cells express endogenous Panx1 (Figure 2A). Panx1 is known to be activated by elevated K + [28,29], which can rise above resting levels as much as 5 mM during periods of intense neuronal activity, up to more than 20 mM during injury and even higher during waves of spreading depression (reviewed in [30]). We treated N2a cells with varying concentrations of extracellular KCl, and found that ATP release was stimulated by elevating KCl concentrations (20 mM) compared to control (5.33 mM) and 0 mM KCl (*P* < 0.01 for one-way analysis of variance (ANOVA) with Tukey *post-hoc*, N = 3) (Figure 2B). Furthermore, treating the cells with the Panx1 blocker probenecid, which selectively blocks Panx1 hemichannels, and not connexin hemichannels [31], significantly decreased ATP release (60.50 ± 7.913% of control, *P* = 0.0159 for non-parametric *t* test) from proliferating N2a cells when compared to controls (Figure 2C). This is in accordance with the reported role of Panx1 in mediating ATP release in numerous other cell types (reviewed in [17]). The remaining ATP release could possibly be mediated by a vesicular release mechanism [32] or by connexin hemichannels ([33], but see also [34]). Finally, blocking Panx1 with probenecid (24 hours: control = 6.9 × 10^4^ ± 6.4 × 10^3^ cells, probenecid = 4.1 × 10^4^ ± 1.8 × 10^3^ cells, *P* < 0.001; 48 hours; control = 1.1 × 10^5^ ± 4.8 × 10^3^ cells, probenecid = 5.6 × 10^4^ ± 4.7 × 10^3^ cells, *P* < 0.001 for one-way ANOVA with Tukey *post-hoc*), or P2 receptors with pyridoxalphosphate-6-azophenyl-2′,4′-disulfonic acid (PPADS) [34], which blocks several P2X and P2Y isoforms [35-42], significantly reduced N2a cell proliferation (24 hours: control = 6.9 × 10^4^ ± 6.4 × 10^3^ cells, PPADS = 4.6 × 10^4^ ± 5.3 × 10^3^ cells, *P* < 0.05; 48 hours: control = 1.1 × 10^5^ ± 4.8 × 10^3^ cells, PPADS = 8.1 × 10^4^ ± 4.4 × 10^3^ cells, *P* < 0.001 for one-way ANOVA with Tukey *post-hoc*) in accordance with the previously described role of P2 receptors in regulating NPC proliferation (Figure 2D) [22-25].

### Panx1 plays a major role in regulating VZ NSC/NPC proliferation

**Figure 3:**
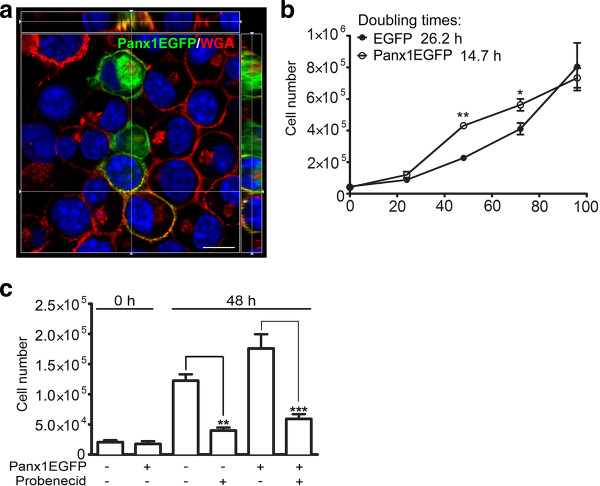
**Panx1 regulates N2a cell proliferation.** (**A**) Image from a confocal z-stack with orthogonal side-views of N2a cells overexpressing Panx1EGFP. Panx1 is highly localized to the plasma membrane, stained with wheat germ agglutinin (WGA), as well as to intracellular membranes. (**B**) N2a cells overexpressing Panx1 exhibited a reduced doubling time (14.7 hours) compared to control cells overexpressing EGFP (26.2 hours) ((*) *P* < 0.05 and (**) *P* < 0.01 for one-way ANOVA with *post-hoc* Tukey’s multiple comparison test, N = 3). (**C**) Treatment of transfected N2a cells with the Panx1 channel blocker, probenecid (1 mM), significantly reduced cell proliferation at 48 hours in both Panx1 and EGFP overexpressing cells ((**) *P* < 0.01 and (***) *P* < 0.001 for one-way ANOVA with *post-hoc* Tukey’s multiple comparison test). ANOVA, analysis of variance; Panx1, pannexin1.

Given that Panx1 mediates ATP release in N2a model NPCs and that blocking P2 purinergic receptors inhibits proliferation, as shown in Figure 2, we hypothesized that Panx1 regulates NSC/NPC proliferation. To investigate the effect of Panx1 on N2a cell proliferation we overexpressed Panx1, and conversely, blocked Panx1 channels with probenecid [31]. Expression of Panx1EGFP, which localizes mainly at the plasma membrane in N2a cells (Figure 3A), as was previously reported in HEK293T cells [43], reduced the doubling time of N2a cells from 26.2 hours to 14.7 hours (cell counts increased by approximately 50%, *P* < 0.01 for one-way ANOVA with Tukey *post-hoc*, at 72 hours *P* < 0.001 for one-way ANOVA with Tukey *post-hoc*, N = 3) (Figure 3B) suggesting a positive effect of Panx1 on proliferation. Similarly, probenecid significantly reduced the proliferation of control EGFP expressing N2a cells, which endogenously express Panx1 (from 1.2 × 10^5^ ± 1.1 × 10^4^ cells to 4.0 × 10^4^ ± 4.9 × 10^3^ cells, *P* < 0.01 for one-way ANOVA with Tukey *post-hoc*, N = 3), as well as Panx1EGFP overexpressing cells (from 1.8 × 10^5^ ± 2.4 × 10^4^ cells to 6.0 × 10^4^ ± 8.0 × 10^3^ cells, *P* < 0.001 for one-way ANOVA with Tukey *post-hoc,* N = 3) (Figure 3C).

**Figure 4:**
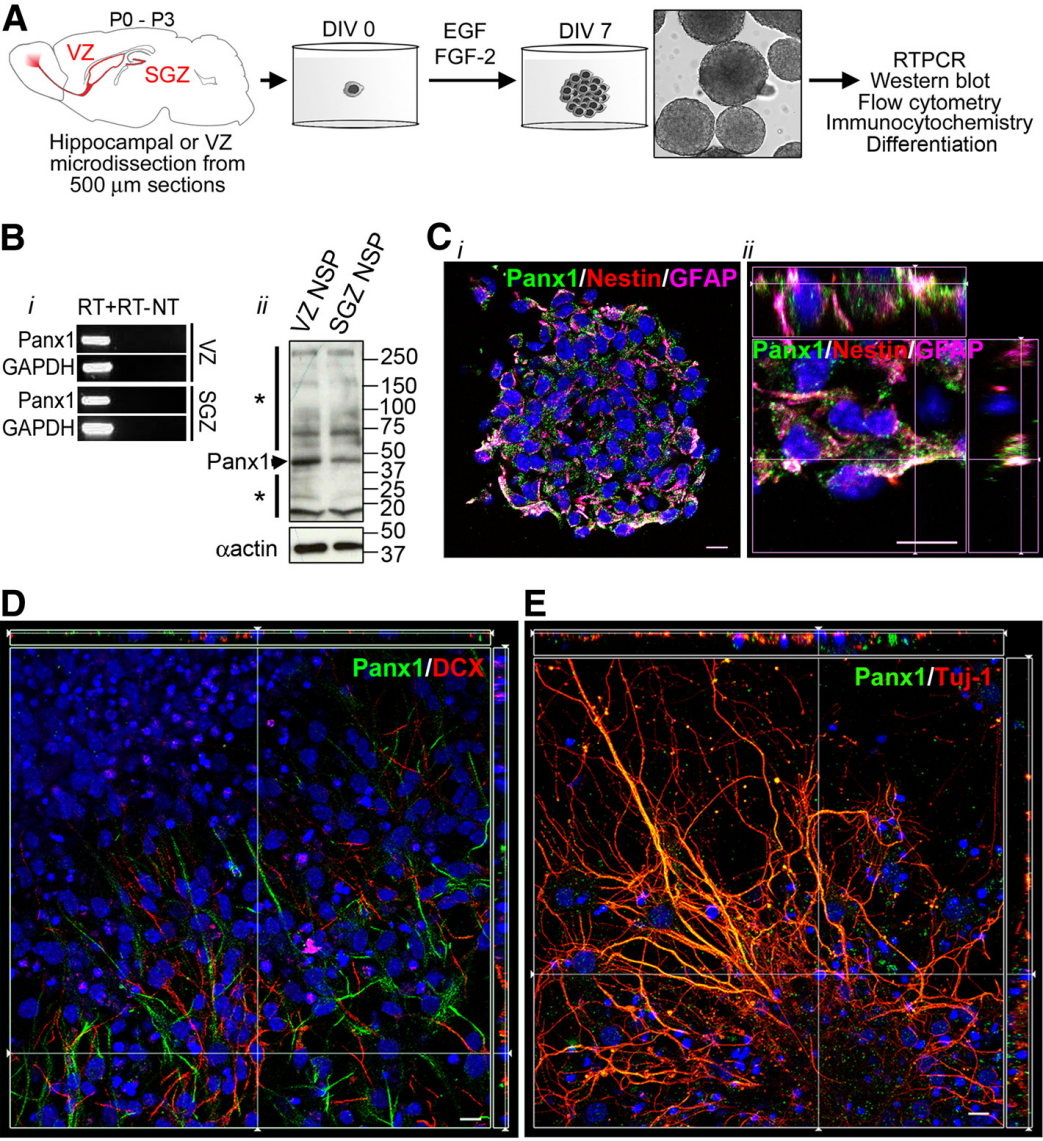
**Panx1 expression in NSC/NPCs is re-capitulated* in vitro* in neurosphere cultures.** (**A**) Outline of neurosphere culture generation from P0 to P3 hippocampus or microdissected VZ. Spheres are cultured for seven days *in vitro* (DIV) with addition of growth factors (hEGF and bFGF) every two DIV, before harvesting for subsequent analyses, or differentiation by removal of growth factors and plating on a poly-D-lysine coated surface. (**B**) Panx1 mRNA (*i*) and protein (*ii*) are expressed in VZ and SGZ neurospheres. To the left of the western blot, the asterisk (*) above the arrow denotes high-molecular weight species likely representing various Panx1 oligomeric forms, while the asterisk below the arrow denotes low molecular weight Panx1 cleavage products consistent in size with those reported in the literature [16]. (**C**) Confocal images showing Panx1 expression in a cryosectioned undifferentiated VZ neurosphere (*i*) and a digitally zoomed z-stack with orthogonal side-views (*ii*) in nestin-positive/GFAP-positive and nestin-positive/GFAP-negative cells. (**D**) Image from a confocal z-stack with orthogonal side-views of Panx1 expression in DCX-negative cells in a differentiated VZ neurosphere. (**E**) Image from a confocal z-stack with orthogonal side-views showing high levels of Panx1 co-expression with Tuj-1 in immature neurons from a differentiated VZ neurosphere. Hoechst 33342 was used as a nuclear counterstain in C-E. Scalebars 10 μm. DCX, doublecortin; GFAP, glial fibrillary acidic protein; NPC, neural progenitor cells; NSC, neural stem cells; Panx1, pannexin1; SGZ, subgranular zone; VZ, ventricular zone.

To determine whether Panx1 also regulates the proliferation of primary NSC/NPCs, we created neurosphere cultures from neonatal mice (P0 to P3), as previously described [10,44] (Figure 4A). Panx1 mRNA and protein were expressed in VZ (and SGZ) derived neurosphere cultures, maintained for seven days *in vitro* (DIV), as assessed by RT-PCR and western blotting (Figure 4B). Confocal immunofluorescence microscopy of VZ neurosphere cultures with lineage markers confirmed Panx1 expression in nestin-positive/GFAP-positive NSCs and nestin-positive/GFAP-negative NPCs (Figure 4C). Furthermore, we plated VZ neurospheres on poly-D-lysine in the absence of mitogenic growth factors to induce differentiation, in order to investigate whether Panx1 is expressed in neuronally committed DCX-positive neuroblasts or Tuj-1-positive immature neurons by confocal immunofluorescence microscopy. As we observed *in vivo*, Panx1 immunoreactivity was absent in DCX-positive neuroblasts cultured under these neuronal driving conditions (Figure 4D). Finally, Panx1 was detected in Tuj-1 positive immature neurons (Figure 4E), consistent with previously reported neuronal Panx1 expression [10,45].

**Figure 5:**
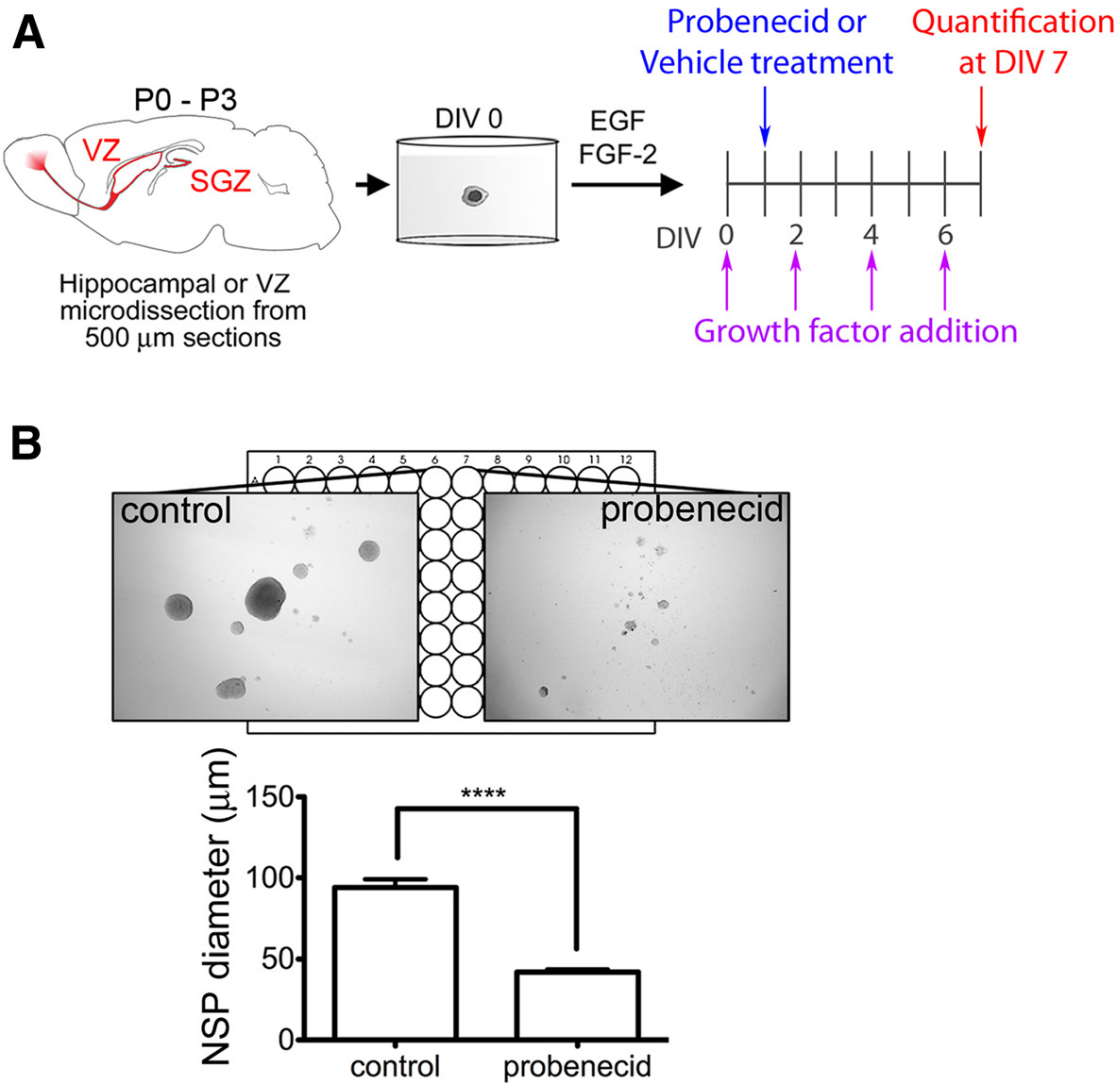
**Panx1 regulates primary NSC/NPC proliferation.** (**A**) Outline of neurosphere treatments *in vitro*. Growth factors (hEGF and bFGF) were added every two DIV beginning at DIV0, while the Panx1 blocker probenecid was added at DIV1 to a concentration of 1 mM. Sphere diameter was measured at DIV7. (**B**) Brightfield images of vehicle and probenecid treated spheres (*i*). A significant reduction in neurosphere diameter was observed following probenecid treatment compared to vehicle treatment (41.85 ± 1.649 μm and 93.97 ± 5.089 μm respectively, *P* < 0.0001 for non-parametric *t* test, N = 12) (*ii*). DIV, days *in vitro*; NPC, neural progenitor cells; NSC, neural stem cells; Panx1, pannexin 1.

We then examined the impact of the Panx1-blocker, probenecid, on the proliferative capacity of VZ neurosphere cultures. Neurospheres were cultured in the absence or presence of 1 mM probenecid from DIV1 onwards. Neurospheres were observed each day by light microscopy and diameter was measured on DIV7 (Figure 5A). Probenecid-treated neurospheres were significantly smaller than controls (41.85 ± 1.649 μm and 93.97 ± 5.089 μm, respective1y, *P* < 0.0001 for non-parametric *t* test, N = 12) (Figure 5B).

## Discussion

Here we report the discovery of Panx1 expression in NSC/NPCs both *in vivo* and *in vitro*. *In vivo*, Panx1 expression was primarily found in luminal type B NSCs and in some sub-ependymal NSCs. As described above, electron microscopy has shown that the ependymal layer lining the ventricles is not entirely contiguous and that a large proportion of type B cells make direct contact with the ventricle either via a large luminal surface or a thin cellular process [3-8]. Panx1 block by probenecid reduced NSC/NPC proliferation, while overexpression of Panx1 increased proliferation. Although the Panx1 blocker probenecid is also known to target organic anion transporters [46,47], the increase in cell proliferation caused by Panx1 overexpression was almost completely blocked by probenecid suggesting that this is a Panx1-specific effect.

As large pore ion and metabolite-permeable channels, Panx1 make ideal integrators of neurogenic microenvironment signaling in the periventricular region. Accordingly, our results demonstrate a role for Panx1 in the regulation of NSC/NPC proliferation, in part, through mediating the release of ATP (or similar nucleotides like ADP and UTP) that subsequently acts on P2 purinergic receptors [22-25]. Here we induced ATP release by stimulating the cells with elevated extracellular potassium (20 mM), and found a significant reduction in this stimulated release upon probenecid treatment. ATP is also basally secreted in bursts from NSC/NPCs. In other cell types, ATP activates Panx1 channels in a P2-receptor-dependent manner [48]. A similar nucleotide (ATP, ADP or UTP)-dependent positive feedback mechanism could account for the increased proliferation observed under Panx1 overexpression, as proliferation is blocked by the P2 receptor blocker PPADS. We did not detect a large increase in basal ATP levels with Panx1 overexpression, but this is not surprising given the high expression of 5′-nucleotidase on the surface of NSC/NPCs [22].

*What additional signals might be involved in triggering ATP release from Panx1 in NSC/NPCs?* Recently an interaction between Panx1 and the actin cytoskeleton was described [43] in BICR-M1R(k) cells, and Panx1 has previously been shown to be activated by cell membrane stretch [18,20]. Multiple cytoskeletal rearrangements occur in cell division and might perpetuate Panx1 mediated ATP release and downstream purinergic receptor signaling, resulting in continued proliferation. Interestingly, we detected cleavage fragments of Panx1 consistent with recently reported Panx1 caspase 3 cleavage that results in constitutive channel opening during apoptosis, and release of ‘find me’ nucleotide signals necessary for the recruitment of phagocytic cells [16]. Our knowledge of the role of caspases has recently expanded from apoptosis to include many other non-apoptotic cellular roles, including cell proliferation and differentiation (reviewed in [49]). It is tempting to speculate that constitutive Panx1 activity generated by caspase proteolytic cleavage might also be relevant to, or necessary for, its role in NSC/NPC proliferation. As PPADS did not reduce proliferation to the same extent as probenecid (Figure 2), the possibility exists that other Panx1 mechanisms may be involved in addition to an ATP/nucleotide release. Using unbiased proteomic methods, we are actively pursuing the identification of Panx1 protein interaction partners specific to NSC/NPC to elucidate further the signaling pathway(s) that regulate Panx1 function in NSC/NPCs.

The expression and functional role of Panx2 in NSC/NPCs was recently described [10]. In accordance with previous studies in heterologous expression systems [50], the current study demonstrates Panx1 has a very different subcellular distribution profile than Panx2 in NSC/NPCs. While Panx2 was mainly found in discrete intracellular structures, Panx1 is more widely distributed and also found at the plasma membrane (Figures 1,3,4). Also, while Panx2 expression was limited to a small subset of cells, Panx1 appears to be abundantly expressed. Furthermore, recent work suggests that the oligomeric structures and pharmacological sensitivities of Panx1 and Panx2 are fundamentally different [51], suggesting that a physical or functional interaction in NSC/NPCs is unlikely.

During embryonic development, the source of ATP release from VZ radial glia has been reported to be connexin 43 hemichannels [52]. Panx1 channels have only recently come into the picture, having been discovered long after the connexins by homology to the invertebrate innexins [12]. Given the relatively high embryonic expression of Panx1 [15] and the data we present here on postnatal Panx1 expression in postnatal periventricular NSC/NPCs, it would be interesting, in future studies, to examine potential crosstalk or overlap between connexin 43 and Panx1 signaling in NSC/NPCs during embryonic development. For example, perhaps there are subpopulations of NSC/NPCs that express either connexin 43 or Panx1, or if co-expressed, are coupled to or regulated by different signaling paradigms. We do not detect Panx1 in postnatal migrating neuroblasts whereas connexin 43 appears to be expressed in these cells [53], and has been shown to play a major role in newborn cell migration from the periventricular region in the embryonic brain [54-56]; this suggests that the roles of Panx1 and connexin 43 are functionally and somewhat physically distinct.

Recent studies highlight the reparative potential of enhancing the proliferation, recruitment and survival of periventricular neuroblasts (reviewed in [57-59]). To eventually harness this potential it is important to understand the factors regulating the behavior of these cells, including the role of Panx1. Panx1 ion and metabolite-permeable channels are known to open in several pathological circumstances, including elevated extracellular potassium [28], ischemia [60] and elevated amyloid beta peptide exposure [21], a pathogenic feature of Alzheimer’s disease (AD). NSCs are highly sensitive to changes in their environment, especially injury and neurological diseases (reviewed in [61,62]). Some of the changes in neurogenesis seen with brain injury, stroke and AD could conceivably involve pathological activation of Panx1 channels in NSC/NPCs. Therefore, understanding the fundamental role of Panx1 channels in NSC/NPCs is of high importance for brain health and disease.

## Conclusions

In this study we have established that Panx1 is expressed in NSC/NPCs where it plays an important role in the positive regulation of cell proliferation. As previous work has focused mainly on the role of Panx1 in neuropathology, this is, to our knowledge, the first demonstration of a normal physiological role of Panx1 channels in the central nervous system. After discovering Panx1 expression in postnatal VZ NSC/NPCs, *in vivo*, we then sought to elucidate its functional role in neuronal development by manipulating Panx1 expression and activity in N2a cells and primary postnatal neurosphere cultures. We demonstrated that Panx1 channels mediated ATP release, which contributes to positive regulation of proliferation through activation of P2 receptors. Moreover, we found that overexpression of Panx1 robustly and significantly increased N2a cell proliferation and that these effects were abrogated by the Panx1 blocker, probenecid. Furthermore, we detected robust Panx1 expression in postnatal VZ neurosphere cultures. As anticipated, blocking Panx1 activity in these primary NSC/NPC cultures significantly reduced cell proliferation. Future studies are now needed to expand our knowledge of the properties of Panx1 channels in NSC/NPCs, including additional possible mechanisms underlying their role in the regulation of proliferation. Understanding the fundamental roles of Panx1 channels in NSC/NPCs is of critical importance given the vital roles that postnatal neurogenesis plays in normal brain health, as well in the progression of neurological diseases and the response to brain injury.

## Methods

### Animals

C57BL/6 mice were used and all procedures were carried out in agreement with the guidelines of the Canadian Council for Animal Care and the University of Victoria Animal Care Committee.

### Cell culture

Primary NSC/NPC cultures were isolated from postnatal day 0 to3 (P0 to P3) C57BL/6 mouse hippocampus and periventricular zone and expanded as neurospheres for seven days *in vitro* (DIV) as described [9,43]. For proliferation assays, neurospheres were treated at DIV1 with 1 mM probenecid (Invitrogen), 30 μM pyridoxal phosphate-6-azophenyl-2′,4′-disulfonic acid (PPADS; Sigma, Oakville, Ontario Canada) or equivalent volumes of sterile water. At DIV7, neurospheres were visualized/photographed using brightfield illumination, and sphere diameters were measured in Photoshop CS5 (Adobe). N2a cells were cultured in DMEM/F12 supplemented with 10% fetal bovine serum (FBS), 100 U/mL penicillin, and 100 μg/mL streptomycin (all obtained from Gibco/Life Technologies, Burlington, Ontario, Canada). Where indicated, N2a cells were transfected using jetPEI reagent (Polyplus transfection/VWR; Edmonton, Alberta, Canada) according to the manufacturer’s protocol, and Panx1 plasmids were a generous gift from Dr. Dale Laird, University of Western Ontario [43,63]. N2a cells were treated at time 0 with 1 mM probenecid or an equivalent volume of sterile water for proliferation assays, in which cells from a minimum of three wells per condition were counted in quintuplicate at the indicated timepoints.

### Reverse transcriptase-polymerase chain reaction (RT-PCR)

Total RNA was isolated and first-strand synthesis and PCR were carried out as described [43] using the following cycling parameters: 94°C for 5 minutes, 35 cycles of 94°C for 30 seconds, 57°C for 50 seconds, and 72°C for 2 minutes, and a final step at 72°C for 7 minutes. Primers were: 5′-CATTGACCCCATGCTACTCC-3′, 5′- TCAGCCACAGAAGTCACAGG-3′ defining a 248 bp Panx1 amplicon, accession# [GenBank: NM_019482.2)] and 5′-TGGTGCTGAGTATGTCGTGGAGT-3′, 5′-AGTCTTCTGAGTGGCAGTGATGG-3′ defining a 292 bp glyceraldehyde-3-phosphate dehydrogenase (GAPDH) amplicon, accession# [GenBank: NM_008084.2].

### Western analyses

Western analysis was performed as described [9,43]. Samples were homogenized in RIPA buffer (10 mM PBS: 150 mM NaCl, 9.1 mM dibasic sodium phosphate, 1.7 mM monobasic sodium phosphate), 1% Nonidet P-40, 0.5% sodium deoxycholate, 0.1% SDS, protease inhibitor cocktail at 1 μL/10^6^ cells (stock: 0.104 mM 4-(2-aminoethyl)benzenesulfonyl fluoride hydrochloride, 0.08 mM aprotinin, 4 mM bestatin hydrochloride, 1.4 mM N-(trans-epoxysuccinyl)-L-leucine 4-guanidinobutylamide, 2 mM leupeptin hemisulfate salt, 1.5 mM pepstatin-A; Sigma-Aldrich) for 30 minutes and centrifuged for 20 minutes at 12,000 rpm to remove debris. For all Western blots, SDS-PAGE was performed under reducing conditions (dithiothreitol (DTT) and β-mercaptoethanol) without heating.

### ATP release assay

N2a cells were plated at a density of 1.8 × 10^4^ cells/cm^2^ 24 hours prior to ATP assay. Media was removed and cells were washed once with Hank’s balanced salt solution (HBSS; 137.93 mM NaCl, 5.33 mM KCl, 1.26 mM CaCl_2_, 0.9 mM MgCl_2_, 5.56 mM D-glucose, 0.441 mM KH_2_PO_4_, 4.17 mM NaHCO_3_, 0.338 mM Na_2_HPO_4_) and pre-incubated for 10 minutes in HBSS with or without the addition of 0.5 mM probenecid. Eighty percent of the pre-incubation medium was removed, and replaced with an equal volume of high KCl HBSS (117.93 mM NaCl, 25 mM KCl, 1.26 mM CaCl_2_, 0.9 mM MgCl_2_, 5.56 mM D-glucose, 0.441 mM KH_2_PO_4_, 4.17 mM NaHCO_3_, 0.338 mM Na_2_HPO_4_) for a final concentration of 20 mM KCl with or without 0.5 mM probenecid, for 10 minutes. Eighty percent of the treatment medium was removed, spun down for 1 minute at 13,500 rpm, and the top portion was analyzed in triplicate for ATP concentration using an ATP determination kit from Molecular Probes/Life Technologies, Burlington, Ontario, Canada). Data are from five independent replicates and were normalized to cell number.

### Confocal microscopy

Neurosphere, P15, P30 and P60 mouse brain cryopreservation, serial cryosectioning (10 μm for neurosphere, 20 μm for brain) were performed as described [10,44,64]. Antibodies were diluted in 10 mM PBS supplemented with 0.3% Triton-X-100 and 3% BSA. Primary antibodies were: rabbit polyclonal anti-Panx1 Cterm (1:40 Invitrogen), rat monoclonal anti-GFAP (1:40, Invitrogen/Life Technologies, Burlington, Ontario, Canada), mouse monoclonal anti-nestin (1:100, Chemicon/Millipore, Temecula, California, USA), guinea pig polyclonal anti-doublecortin (DCX; 1:800, Chemicon/Millipore), mouse monoclonal anti-Tuj1 class III β-tubulin (1:250, RDI). Secondary antibodies were Cy3-conjugated anti-rabbit IgG (1:600), DyLight488-conjugated anti-rabbit IgG (1:100), Cy3-conjugated anti-guinea-pig IgG (1:800), DyLight488-conjugated anti-mouse IgG (1:600), and DyLight649-conjugated anti-mouse IgG (1:400). Hoechst 33342 (1 μg/mL) was used as a nuclear counterstain. To label N2a cell membranes, cells were treated with 5 μg/mL TRITC-conjugated wheat germ agglutinin (WGA, Molecular Probes/Life Technologies) for 5 minutes at 37°C in HBSS (137 mM NaCl, 5.4 mM KCl, 0.25 mM Na_2_HPO_4,_ 0.44 mM KH_2_PO_4,_ 4.2 mM NaHCO_3_; Gibco/Life Technologies), washed in HBSS, fixed in 3.7% paraformaldehyde for 15 minutes and processed for immunostaining. Confocal immunofluorescence imaging was performed on a Zeiss LSM 700 confocal microscope and images were captured sequentially with ZEN 2009 software on an EC Plan-Neofluar 40x/1.30 oil DIC M27 objective with equal optical slices for multiple fluorophores (approximately 1 Airy Unit) through z-stacks in 0.35 μm optical sections.

### Statistical analyses

Significance was determined using Student’s *t* tests or ANOVA with Tukey multiple comparisons *post-hoc* test. Variances are reported as standard error of the mean.

## Abbreviations

AD, Alzheimer’s disease; ANOVA, analysis of variance; bFGF, basic fibroblast growth factor; BSA, bovine serum albumin; DIV, days in vitro; DTT, dithiothreitol; DCX, doublecortin; DMEM/F12, Dulbecco’s modified eagle medium/F12; EGFP, enhanced green fluorescent protein; FBS, fetal bovine serum; GFAP, glial fibrillary acidic protein; GAPDH, glyceraldehyde-3-phosphate dehydrogenase; HBSS, Hank’s buffered salt solution; HEK293T cells, human embryonic kidney 293T cells; hEGF, human epidermal growth factor; NPC, neural progenitor cell; NSC, neural stem cell; N2a, Neuro2a; Panx, pannexin; PBS, phosphate buffered saline; P, postnatal day; PPADS, pyridoxalphosphate-6-azophenyl-2′,4′-disulfonic acid; RT-PCR, reverse-transcriptase polymerase chain reaction; SGZ, subgranular zone; Tuj-1, class III beta tubulin; UTP, uridine triphosphate; V, ventricle; VZ, ventricular zone; WGA, wheat germ agglutinin.

## Competing interests

The authors declare that they have no competing interests.

## Authors’ contributions

LWS and LAS planned the experiments, prepared the figures and wrote and revised the manuscript. LWS performed all tissue culture and transfections associated with N2a cells and primary neurospheres. ADD, JMS and RMS also assisted in planning selected experiments. LWS and ADD performed cell growth/proliferation assays and ATP assays. RMS performed RT-PCR and cryosectioning of neurosphere and brains. LWS and JMS performed confocal immunofluorescence microscopy experiments and western blotting. All authors read and approved the final manuscript.

## Authors’ information

LAS is an Assistant Professor in the Division of Medical Sciences at the University of Victoria.
